# Early-life exposure to di (2-ethyl-hexyl) phthalate: Role in children with endocrine disorders

**DOI:** 10.3389/fcell.2023.1115229

**Published:** 2023-02-10

**Authors:** Fa Zeng, Luodan Zhang, Fang Deng, Shuiping Lou

**Affiliations:** ^1^ Shenzhen Longhua Maternity and Child Healthcare Hospital, Shenzhen, China; ^2^ Department of Nephrology, Anhui Provincial Children’s Hospital, Children’s Hospital of Anhui Medical University, Hefei, China

**Keywords:** di (2-ethyl-hexyl) phthalate (DEHP), endocrine-disrupting chemicals (EDCs), early-life, placenta, offspring, lipid and glucose

## Abstract

Di (2-ethyl-hexyl) phthalate (DEHP), one of endocrine-disrupting chemicals (EDCs), has widespread concern due to its serious health hazards. Exposure to DEHP in the early stage of life affects fetal metabolic and endocrine function, which even would cause genetic lesions. To date, it is widely believed that the increasing incidence of childhood obesity and diabetes in adolescents is related to the impact of DEHP on glucose and lipid homeostasis in children. However, there remains a knowledge gap to recognize these adverse effects. Thus, in this review, besides the exposure routes and levels of DEHP, we further outline the effects of early-life exposure to DEHP on children and potential mechanisms, focusing on the aspect of metabolic and endocrine homeostasis.

## 1 Introduction

Di (2-ethyl-hexyl) phthalate (DEHP), belonging to the family of phthalates, is a plasticizer and solvent in polyvinyl chloride (PVC), which can be used to manufacture various products, such as cosmetics, toys, and medical tubing (Li et al., 2012). Through the widespread use of plastic products, phthalates would migrate to other products or the environment, indirectly or directly affecting human health (Cho et al., 2015). As endocrine-disrupting chemicals in the external environment, long-term exposure would have adverse effects on human health (Stojanoska et al., 2017). It is noted that DEHP shows obvious reproductive toxicity (Zhang et al., 2020) and results in much more problems, such as ovulation disorders, precocious puberty, and abnormal pregnancy. Endocrine and metabolic disorders have become risk factors for pregnancy complications, diabetes, and obesity (Huang et al., 2020; Zhang et al., 2022).

According to DOHaD theory, which is based on an epidemiological study about low birth weight and malnutrition (Barker, 2007), the effects of exposure before and during pregnancy would be reflected in the health status of offspring. If it is affected in the period of early life, it will interfere with the original growth process, resulting in incorrect coding and expression The DOHaD theory is now widely used in research on diseases, especially chronic diseases. This early-life nutritional theory is considered applicable to most chronic diseases. For organisms, the intrauterine period is the key phase of most organ development (Roseboom et al., 2001; Barker, 2007; Agarwal et al., 2018). In researches on chronic diseases, researchers often use the experimental method of maternal intervention during pregnancy to determine whether the research factors would produce the corresponding outcome by observing the performance of the offspring. DEHP exposure in early life can damage the endocrine system of offspring (Qian et al., 2020). DEHP could affect the fetal reproductive system by regulating the synthesis of hormones during pregnancy (Liu et al., 2021a). In female offspring, DEHP exposure may regulate ovarian hormone production, thereby affecting the development of follicles (Liu et al., 2017; Liu et al., 2021b). Additionally, low-dose DEHP exposure is recognized as a potential risk factor for obesity and metabolic syndrome in offspring during early life stages (Fan et al., 2020). A prospective cohort study shows that maternal exposure to phthalates may affect glucose and lipid metabolic disorders, with potential persisting sex specificity in childhood (Sol et al., 2020). Understanding the detailed mechanisms that control endocrine metabolism is vital for improving the health of children.

Thus, in this review, we summarize the effects of early-life exposure to DEHP on endocrine homeostasis in offspring and the potential mechanism, and highlight the common connections existing in the current research, providing some insights for further scientific research and medical precaution.

## 2 Exposure routes and levels of DEHP

As general endocrine-disrupting chemicals, phthalates are common additive in the plastic manufacturing industry and are mainly used to increase the ductility, elasticity, and strength of plastic products, including food package, plastic utensils, agricultural plastic film, and medical PVC devices (Giuliani et al., 2020). As the combination of phthalates and the main body of the plastic matrix is non-covalent, phthalates would migrate out of various products and dissolve into the external environment, causing environmental pollution and affecting human health (Heudorf et al., 2007; Bolling et al., 2020). People are generally exposed to phthalates through soil pollution and air pollution in the external environment. Phthalates can enter the human body through the respiratory system, digestive system, and skin. Among them, absorption *via* the digestive tract through food intake is the largest intake route of phthalates (Wang et al., 2019). Besides, dust ingestion is also major exposure route of phthalates, with dosage in the range from 1.12 μg/kg in infants to 1.7 μg/kg in toddlers (Tran and Kannan, 2015). Table 1 shows the concentrations of various phthalates metabolites, including MEP, MBzP, MEHP, MEHHP, MEOHP and MECPP from DEHP, and MnBP, MIBP, MCPP in parent and child urine samples, respectively (Tellez-Rojo et al., 2013; Wu et al., 2017).

**TABLE 1:** Concentrations of various phthalate metabolites in urine (SG-corrected, ng/mL).

Source of sample	Phthalate metabolite (ng/ml)									References
	MEP	MnBP	MIBP	MBzP	MCPP	MEHP	MEHHP	MEOHP	MECPP	
Mother	138	85.61	2.30	3.54	1.75	6.56	22.08	14.23	39.65	Tellez−Rojo et al. (2013)
Children	30.8	61.2	17.2	2.90	5.8	10.4	40.66	10.8	12.6	Wu et al. (2017)

## 3 Effects of early-life exposure to DEHP on endocrine homeostasis in children

### 3.1 Early-life exposure to DEHP could cause childhood obesity

Childhood obesity has a great impact on health in adulthood, while adolescent obesity can also lead to psychological problems. People who were overweight or obese as children or adolescents are more likely to continue this trait in adulthood, and childhood obesity is associated with a variety of adverse outcomes (Di Cesare et al., 2019). A cohort study of 4,857 American Indian children without diabetes evaluated the association of body mass index (BMI), blood pressure level, cholesterol content, and premature death (Franks et al., 2010). Moreover, obesity in children and adolescence may be related to cardiovascular disease, diabetes, and various other causes (Bendor et al., 2020). These findings suggest that preventing obesity in the early stages of life is of great significance to health for the whole life cycle.

Increasing research on environmental endocrine disruptors has revealed that endocrine disruptors in the external environment are closely related to human glycolipids. A variety of established or potential environmental factors, including phthalate, polychlorinated biphenyls (PCBs), and perfluoroalkyl acids, can contribute to glucose and lipid metabolism in the body (Newbold et al., 2009; Do et al., 2017; Veiga-Lopez et al., 2018). Environmental factors can have an impact from the early stages of human life, and research in this field can help analyze the causes of obesity.

Perinatal exposure to DEHP may increase the incidence of obesity in offspring and DEHP may be a potential chemical stressor of obesity and obesity-related diseases (Hao et al., 2013). A meta-analysis summarizing original papers on the association between phthalate exposure and obesity in children and adults up to 2019 shows that there is an association between DEHP and adult obesity in general, but it was not conclusive (Ribeiro et al., 2019). Furthermore, the relationship between DEHP and childhood obesity has also been controversial in previous studies (Ribeiro et al., 2019). In a cohort study, the concentration of phthalates metabolites in urine collected twice during pregnancy is positively correlated with height, weight, waist circumference, body fat percentage, and other physical indicators of children aged 5–12 years (Harley et al., 2017). A cohort study of African American pregnant women shows that prenatal exposure to phthalates is associated with a lower BMI in early childhood (Maresca et al., 2016). In a cross-sectional study, an analysis of baseline data from 1,239 American girls between the ages of 6 and 8 years indicated that there is a weak but measurable relationship between phthalate exposure and BMI and waist circumference (Deierlein et al., 2016). A survey of middle-aged mothers shows that DEHP exposure before birth has a greater impact on the weight of male offspring, suggesting that prenatal DEHP exposure would affect the birth weight of the fetus and that there are gender differences in this effect (Zhang Y. W. et al., 2018b; Shafei et al., 2018). Collectively, these reports indicated that early-life exposure to DEHP could cause energy metabolism disorder, thereby disrupting endocrine homeostasis in children.

### 3.2 Early-life exposure to DEHP could disrupt endocrine homeostasis in children

In animal experiments involving metabolomics evaluation, the weight of offspring significantly increased after exposure to DEHP during pregnancy in mice, and the performance of the male offspring at low doses of DEHP is more obvious (Hao et al., 2013). Meanwhile, the liver metabolism of offspring is impaired in childhood, and glucose and lipid homeostasis are markedly abnormal (Hao et al., 2012; Rajagopal et al., 2019). These results suggest that maternal exposure to phthalates impairs liver function and metabolism in the offspring. However, a more detailed mechanism still needs to be elucidated.

Exposure to DEHP during pregnancy is associated with shortening of the pregnancy cycle, suggesting that exposure to DEHP during pregnancy can significantly increase the risk of preterm birth (Yu et al., 2018; Ferguson et al., 2019). It is reported that DEHP could cross the placental barrier and cause premature birth before 37 weeks. Infants whose gestational age is lower than MEHP negative (Huang et al., 2014). These studies indicate that the adverse effects of DEHP on fetal growth parameters may partly depend on the reduction of gestational age, rather than the direct effects of phthalates, since the gestational age of the fetus is also related to fetal growth parameters (Huang et al., 2014). In contrast, there are reports that DEHP exposure during pregnancy may have no physiological effects on the fetus. In a study of 404 multiethnic women in late pregnancy in New York, the establishment of phthalate biomarkers and multivariate adjustment model analysis reveal that DEHP-MWP and high MWP metabolites are associated with any birth outcome with no significant correlation (Patti et al., 2021).

## 4 Potential mechanisms of early-life exposure to DEHP affecting offspring health

**FIGURE 1:**
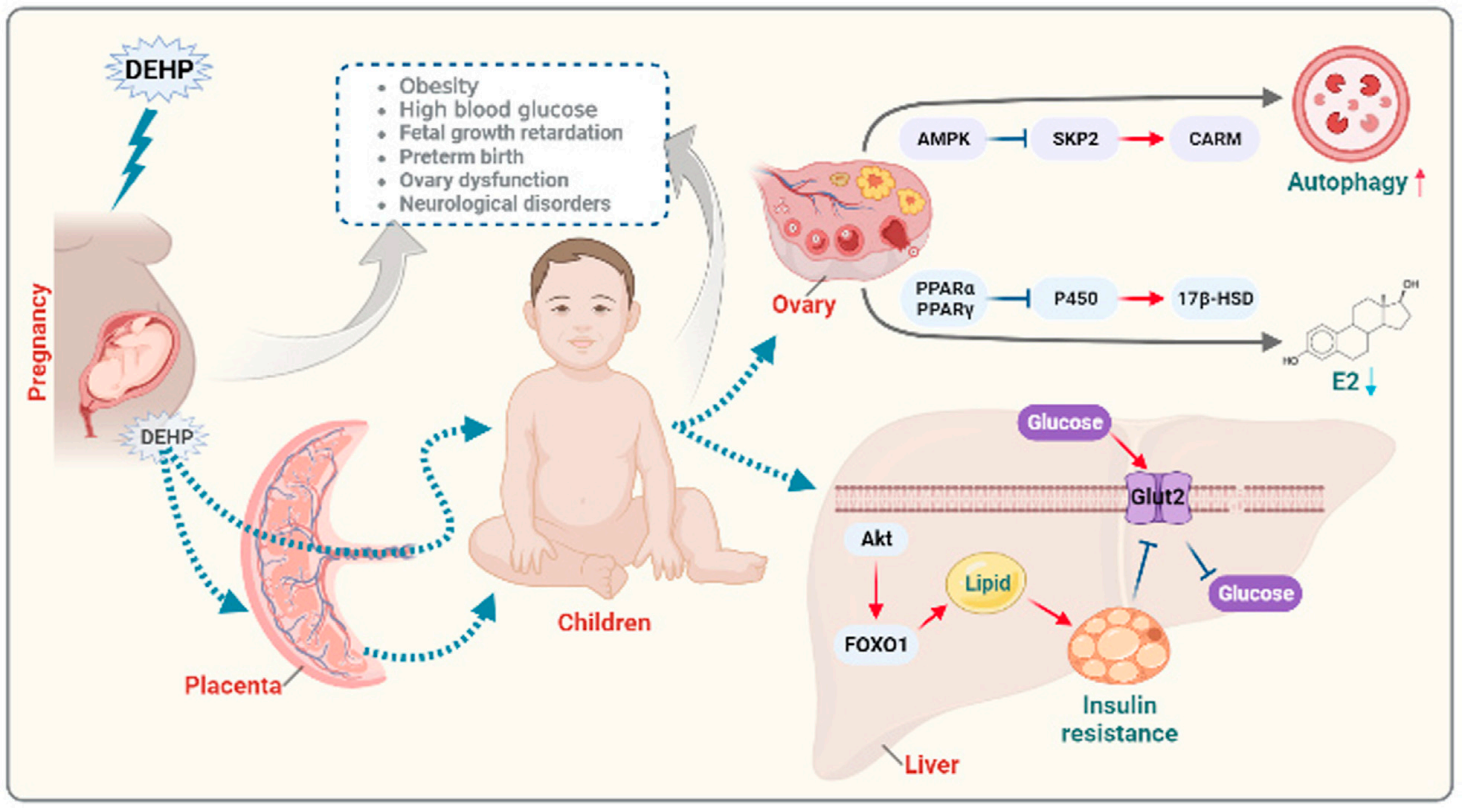
Diagram of the potential mechanisms of prenatal exposure to DEHP in offspring endocrine disorders. Functionally, DEHP could cross the placental barrier and directly or indirectly act on the fetus leading to growth retardation, preterm birth, ovary dysfunction and neurological disorders. Maternal exposure to DEHP could induce obesity and high blood glucose in offspring. Mechanically, maternal exposure to DEHP could activate AMPK-SKP2-CARM signaling to disrupt follicular development *via* autophagy in the offspring ovary. Moreover, DEHP can decrease P450 aromatase by activating PPARα and PPARγ, leading to the inhibition of ovarian secretion of hormones. Maternal DEHP exposure involving in regulating GLUT2 expression and in the liver leading to insulin resistance through FOXO1 signaling in immature male offspring. The figure was created with BioRender.com.

DEHP not only directly affect the exposed population but also affect the offspring of the exposed population. The DOHaD theory proposes that there is a critical period of growth and development early in life, which is an important stage of fetal gene coding and expression. If environmental factors interfere with gene expression during this period, then the growth trajectory of life can be changed (Suzuki, 2018). DEHP has been reported to cross the placental barrier and directly or indirectly act on the fetus. The effect of DEHP on development is already in progress during the period of intrauterine growth (Latini et al., 2003). Maternal exposure to DEHP could activate AMPK-SKP2-CARM1 signaling to disrupt follicular development *via* autophagy in the fetal ovary in a mouse model (Zhang Y. et al., 2018a). Moreover, exposure of suckling mice to DEHP during lactation could affect hormone production, which is involved in the development of follicles, through the oxidative stress pathway (Liu et al., 2021a). Previous studies have shown that phthalates can decrease P450 aromatase by activating PPARα and PPARγ, leading to the inhibition of ovarian secretion of hormones (Lovekamp-Swan and Davis, 2003; Ito et al., 2019; Li et al., 2020; Meling et al., 2022). Early-life exposure to phthalates is a potential risk factor for obesity and metabolic disorders in offspring (Fan et al., 2020). Maternal DEHP exposure also plays a significant role in metabolic disorders by regulating GLUT2 expression and epigenetics in the liver, which are involved in insulin resistance in immature male rat offspring (Rajagopal et al., 2019). Exposure to DEHP might induce glucose metabolic disorder in offspring through the JAK2/STAT3/SOCS3 pathway, which is involved in regulating insulin and leptin signaling pathways (Xu et al., 2018). In addition, DEHP was recently reported to promote the overexpression of FOXO1 to induce insulin resistance and hepatic lipid accumulation (Wei et al., 2022). An overview of the mechanism how exposure to DEHP affect offspring health is summarized in Figure 1.

## 5 Conclusion and perspectives

Existing research suggests there is a strong correlation between maternal exposure to DEHP and childhood obesity, in accord with the results observed in animal experiments. However, the exposure dose of DEHP s in each study was not identical, and the exposure dose of DEHP in animal experiments differs from the human exposure dose. Compared with rodents, primates are less sensitive to DEHP and this has been attributed to differences in the absorption, distribution, metabolism, and excretion of DEHP between these mammals (Matsumoto et al., 2008). Consequently, there may be some differences between the experimental results obtained in rodents and the actual situation in humans. Combining human and animal studies from a more specific molecular level would facilitate to explore the effects of DEHP exposure on endocrine metabolism in offspring. Evaluation of the clinical significance of DEHP exposure is difficult in epidemiological studies. Increasing studies that DEHP can cause changes in physiological functions analyze the effect of DEHP at the molecular mechanism level. Future work should also focus on related research in the field of epigenetics to explain the impact of DEHP on offspring.
